# Two-state model explaining thermodynamic regulation of thermo-gating channels

**DOI:** 10.52601/bpr.2022.220012

**Published:** 2022-08-31

**Authors:** Xuejun C. Zhang, Zhuoya Yu

**Affiliations:** 1 National Laboratory of Biomacromolecules, CAS Center for Excellence in Biomacromolecules, Institute of Biophysics, Chinese Academy of Sciences, Beijing 100101, China; 2 College of Life Sciences, University of Chinese Academy of Sciences, Beijing 100049, China

**Keywords:** Temperature sensing, Thermo-TRPs, Thermo-gating channel, Two-state model, Differential conformation energy

## Abstract

Temperature-sensitive ion channels, such as those from the TRP family (thermo-TRPs) present in all animal cells, serve to perceive heat and cold sensations. A considerable number of protein structures have been reported for these ion channels, providing a solid basis for revealing their structure–function relationship. Previous functional studies suggest that the thermosensing ability of TRP channels is primarily determined by the properties of their cytosolic domain. Despite their importance in sensing and wide interests in the development of suitable therapeutics, the precise mechanisms underlying acute and steep temperature-mediated channel gating remain enigmatic. Here, we propose a model in which the thermo-TRP channels directly sense external temperature through the formation and dissociation of metastable cytoplasmic domains. An open–close bistable system is described in the framework of equilibrium thermodynamics, and the middle-point temperature *T*_½_ similar to the *V*_½_ parameter for a voltage-gating channel is defined. Based on the relationship between channel opening probability and temperature, we estimate the change in entropy and enthalpy during the conformational change for a typical thermosensitive channel. Our model is able to accurately reproduce the steep activation phase in experimentally determined thermal-channel opening curves, and thus should greatly facilitate future experimental verification.

## INTRUDUCTION

### Versatile TRP family channels

Temperature-gating ion channels constitute the primary sensors in neurons responsible for rapidly detecting temperature changes, thus protecting animals from potentially lethal temperature conditions (Clapham 2003; Samanta* et al.* 2018). Well-studied thermo-gating ion channels include the heat-sensitive channel TRPV1 (Caterina* et al.* 1997), which can also be activated by capsaicin, and the cold-sensitive channel TRPM8 (McKemy* et al.* 2002; Peier* et al.* 2002), which can also be activated by menthol. Both channels belong to the family of transient receptor potential (TRP) channels, which is named after its homology to the* trp* gene product of *Drosophila* (Wes* et al.* 1995), and which is conserved from nematodes to humans. TRP channels comprise a class of cationic channels that convert environmental stimuli to cellular signals in the form of alterations of membrane potential and/or intracellular calcium (Samanta* et al.* 2018). To date, 28 TRP channels with versatile properties have been identified in mammals, and they are phylogenetically divided into six subfamilies. Six of those TRP channels are known to perceive temperature, and are thus specifically termed temperature-sensitive TRPs or thermo-gating TRPs (thermo-TRPs). Members of the TRP family (including those from different subfamilies) and their splice variants generated during the transcription process can form either homo- or hetero-tetramers, further expending potential TRP channels and providing the cells of our bodies with tailored responses to a variety of cellular and environmental stimuli (Samanta* et al.* 2018).

TRP channels are also known as polymodal channels as they commonly respond to multiple external stimuli (Benitez-Angeles* et al.* 2020). A variety of TRP channels serve to perceive temperatures (*e*.*g*., > 43 °C), inflammatory pain, osmotic stress, environmental acidity (*e*.*g*., pH < 5.9), and various irritating compounds and, in response, mediate membrane permeability of cationic ions (especially Ca^2+ ^). Studying the regulatory mechanisms of TRP, including a synergy or mutual inhibition between various stimuli, may advance the identification of compounds critical for analgesia, anesthesia, and other medical fields.

### Architecture of TRP channels

**Figure 1:**
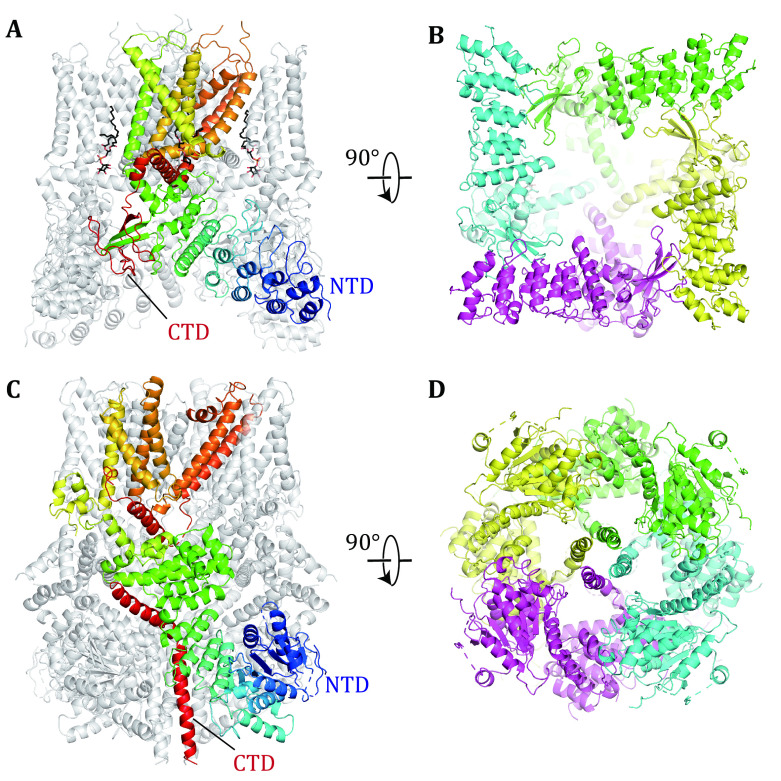
Overall structures of TRPV1 and TRPM8. Side (**A**) and bottom (**B**) view of the cryo-EM structure of TRPV1 (PDB ID: 7LQY). Side (**C**) and bottom (**D**) view of the cryo-EM structure of TRPM8 (PDB ID: 6O6A). In panel A, phosphatidylinositol (PtdIns) molecules are shown as black sticks. In panels A and C, a single subunit is colored by a rainbow scheme (N-terminal blue and C-terminal red), and others are grey. In panels B and D, the four subunits are displayed in distinct colors. TRPV1 and TRPM8 are clearly of distinct cytosolic domains

Research on agonists and antagonists of thermo-TRPs has received extensive attention in an effort to develop novel pain-controlling substances (Benitez-Angeles* et al.* 2020). Currently, structures of TRPV1 are widely studied to identify binding modes of a variety of modulators (Cao* et al.* 2013; Gao* et al.* 2016; Liao* et al.* 2013; Yang* et al.* 2015). The TRPV1 channel is a prototypic member of the TRP family and functions as a homo-tetrameric complex, with an overall structure similar to that of the classical tetrameric voltage-gating channels. Each subunit contains an intracellular N-terminal domain (NTD, containing six ankyrin repeats), a six-helices transmembrane domain (TMD, S1−S6), and a C-terminal domain (CTD, containing a TRP box) (Fig. 1A and 1B). NTD is slightly larger than CTD and wraps around the tetramer formed by C-terminal domains. Together, both terminal domains from each of the four subunits form a cytosolic oligomeric domain, composing ~70% of the total mass of the channel complex. Similar to those in a typical tetrameric ion channel, in TMD of each TRP subunit, S1−S4 form a voltage sensor-like domain (VSD), whereas S5, S6, and a re-entrant pore loop in between participate in the formation of the central pore of the channel complex. Intriguingly, the S4 helix from VSD in TRP is no longer decorated with multiple positive charges, whereas the TRPV1 channel complex maintains its voltage-gating ability (Voets* et al.* 2004). Furthermore, the interfaces between VSDs and the central pore have evolved to become binding sites for multiple agonists (such as capsaicin and cactus toxin) (Yang* et al.* 2015). The binding of the agonist presumably pushes on the amphiphilic helix connecting S4 and S5, which in turn causes the opening of the nearby central gate. Interestingly, the binding of two capsaicin molecules is sufficient to activate the tetrameric TRPV1 channel (Liu* et al.* 2019).

In the apo structure of TRPV1 (PDB ID: 7LQY), the capsaicin-binding pockets are each occupied by a phosphatidylinositol (PtdIns) molecule (Fig. 1A) (Gao* et al.* 2016). It is currently assumed that these lipid molecules hinder the opening of the channel at lower temperatures, and that a rising temperature promotes the dissociation of these bound lipid molecules, leading to channel opening. In agreement with this hypothesis, the ratio of various PtdIns derivatives in the cell membrane changes with temperature, which may tune the functions of various membrane proteins through specific binding. However, such a hypothesis presumably represents an over-interpretation of these structural observations. In particular, biochemical reactions during lipid metabolism may not be sufficiently fast to account for the rapid response of the thermo-TRP channel to temperature change. A general and comprehensive mechanism of thermo-gating remains to be established firmly.

The temperature response of TRP channels is shown to be primarily determined by the properties of their CTDs. For instance, when the CTDs of TRPV1 and TRPM8 are swapped, the thermo-sensing properties of those chimeras are also mutually exchanged (Brauchi* et al.* 2006). Both NTDs and CTDs of TRPV1 are significantly different from their counterparts of TRPM8 (Yin* et al.* 2018), and these structural differences are likely responsible for their functional differences. In addition, phosphorylation in CTD and protonation of conserved acidic residues on certain extracellular loops potentiate the activity of TRPV1 (Jordt* et al.* 2000). As part of the negative feedback regulation, the C-terminal domain also contains a binding region for calmodulin, which will inhibit channel activity upon the increase of intracellular Ca^2+ ^ concentration (Numazaki* et al.* 2003). A variety of long-chain unsaturated fatty acids (such as ω-3 fatty acids) can also activate the TRPV1 channel, with a binding mode presumably similar to capsaicin (Matta* et al.* 2007). These earlier findings strongly indicated that thermo-TRP channels behave in many ways similar to other less-exotic channel families. On the basis of these observations, two important questions arise, namely what the physical basis for the thermo-gating ability of thermo-TRPs is, and whether it is possible to delineate a temperature-gating mechanism similar to that of voltage-gating channels?

## MECHANISM OF TEMPERATURE-MEDIATED GATING OF TRP CHANNELS

At least three gating mechanisms for thermo-gating channels have previously been proposed (Clapham 2003): (1) Changes in the affinity of ligands (*e*.*g*., certain secondary messengers) triggered by temperature changes; (2) changes in membrane tension with temperature; and (3) conformational change of the channel itself with temperature variation. However, both the first and second hypothetical mechanisms are difficult to reconcile with the observed steep activation phase in the experimentally determined temperature-channel opening curve (Voets* et al.* 2004). Moreover, a variety of thermo-TRPs, which physiologically function in different cell types and respond to distinct temperature ranges, have been heterologously expressed in the same model cells (and even in cell-free membrane patches) and shown to function normally (Voets* et al.* 2004). These results strongly suggest that thermo-gating constitutes an intrinsic property of the channel rather than depending on specific factors from the host cells. Furthermore, in thermal stability studies (such as ThermoFluor analysis (Cummings* et al.* 2006)) of protein samples (including membrane proteins), a globular protein domain or a spherical complex tends to denature at a specific melting temperature (*T*_m_), thereby losing its three-dimensional conformation in a phase transition-like manner. This type of general observations in protein thermostability inspired us to explore the above-mentioned third mechanism and to formulate its thermodynamic details.

One widely-accepted thermodynamic mechanism of channel gating is described by the bistable model (Zhang* et al.* 2018). According to this model, the close and open conformations of the channel represent two thermodynamic states, which differ in their Gibbs free energy by the differential conformation energy term Δ*G*_C_. Since we are interested in assessing the fast process of channel opening, the slow inactivation (or desensitization) process can be omitted from the current discussion. With such an assumption, we can describe thermo-gating using the following equation:

1 $ \Delta {G}_{\mathrm{C}}={G}_{\mathrm{o}\mathrm{p}\mathrm{e}\mathrm{n}}-{G}_{\mathrm{c}\mathrm{l}\mathrm{o}\mathrm{s}\mathrm{e}}=\mathrm{ }\Delta H-T\Delta S , $

where Δ*H* and Δ*S* are the enthalpy- and entropy-change associated with the channel opening, respectively. In thermodynamics, a pair of variables like temperature (*T*) and entropy (Δ*S*) are considered conjugated; they behave in a similar way to the charge–voltage (Δ*Q*–*V*) pair and section area–membrane tension (Δ*A*–*σ*) pair, among many others. At a given temperature (*T*), the opening probability *P*_open_(*T*) is defined by the following Boltzmann distribution:

2 $ {P}_{\mathrm{o}\mathrm{p}\mathrm{e}\mathrm{n}}\left(T\right)=\left[1 + \mathrm{e}\mathrm{x}\mathrm{p}\left(\frac{{\Delta G}_{\mathrm{C}}\left(T\right)}{RT}\right)\right]^{-1}. $

**Figure 2:**
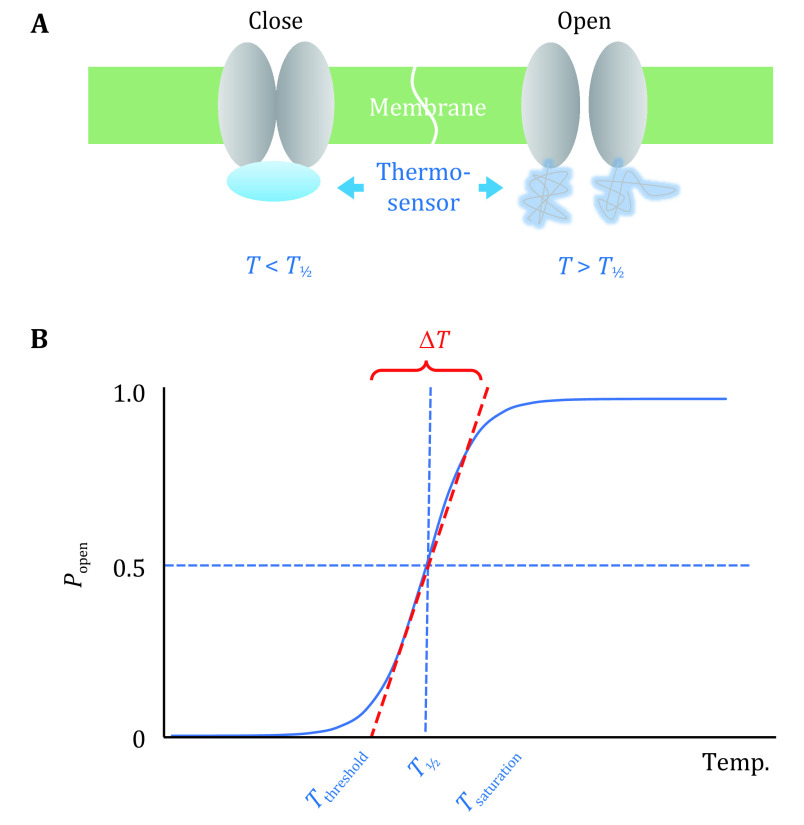
Two-state model of the type-I temperature-mediated channel gating. **A** Schematic diagram of the thermo-gating mechanism. **B** Probability of channel opening as a function of temperature for a bistable, type-I, hyperthermic thermo-gating ion channel

The more negative is Δ*G*_C_, the more likely the channel is present in its open state. Since Eq. 2 includes the variable *T*, the opening probability of the channel should naturally respond to temperature changes. However, how temperature variation induces a thermo-gating channel to produce the observed phase transition-like *P*_open_(*T*) curve while approaching its threshold temperature (*T*_thr_, the estimated starting point for the channel opening; Fig. 2), is a critical question that we want to address here.

Similar to the above-mentioned melting temperature (*T*_m_) in protein folding, we define the middle-point temperature, *T*_½_, at which *P*_open_ equals 0.5, *i.e.,* Δ*G*_C_ equals zero. As shown in Fig. 2, *T*_½_ is a thermodynamic parameter that is more accurately defined than *T*_thr_. The presence of a well-defined *T*_½_ would indicate that during the thermo-gating process, the channel complex undergoes cooperative conformational changes, accompanied by significant changes in both Δ*H* and Δ*S*. In other words, in the vicinity of *T*_½_, the marginal thermal stability exhibited by the channel complex results from a compromise between the enthalpy which promotes stabilization of the thermo-sensor, and the entropy which promotes dissociation or unfolding of the sensor.

In a polymodal channel, ligand binding and/or change of the transmembrane voltage are found to affect the threshold temperature (*T*_thr_); conversely, temperature change shifts the voltage–*P*_open_ curve by affecting the middle point voltage (V_½_) (Voets* et al.* 2004). These observations suggest that a change in temperature alters the differential free energy of conformational change (Δ*G*_C_) during channel opening in a yet to-be-defined manner, resulting in synergistic activation with other regulatory factors. In other words, Δ*G*_C_ coordinates various physical factors that affect channel gating in a pivotal manner. In case that the opening of the channel corresponds to a larger increase in entropy (*i.e.,* Δ*S* > 0; such as the dissociation of the cytosolic domain oligomers), increasing the temperature results in a decrease in Δ*G*_C_, ultimately resulting in the opening of the channel. Here, we define such a temperature rising-mediated channel opening model as the type-I thermo-gating mechanism. Conversely, if the opening of the channel corresponds to a significant decrease in entropy (*i.e.*, Δ*S* < 0; such as stabilization of the cytosolic domain complex), lowering the temperature will result in a decrease in Δ*G*_C_ as well as the opening of the channel. Here, we termed such a temperature reduction-mediated channel opening model as the type-II thermo-gating mechanism.

Similar to the proportional relationship between the gating charge and the slope of the opening probability curve of a voltage-gating channel, the larger the magnitude of Δ*S*, the more step-function the *P*_open_(*T*) curve likes. Thus, the curve at *T*_½_ will become steeper with an increase of |Δ*S*|, and *T*_thr_ may be estimated more accurately from a measured *P*_open_(*T*) curve. We propose that in the absence of a sophisticated positive feedback circuit, utilizing a larger entropy change represents the only reliable way to achieve a steep temperature sensitivity. Under experimental conditions, in which all non-temperature external factors are fixed, *T*_½_ equals to the constant ratio of Δ*H*/Δ*S*. This ratio essentially represents the melting temperature (*T*_m_) of the thermo-sensor, and is a characteristic property of the thermo-gating channel. Similar to the V_½_ parameter of a voltage-gating channel, a linear relationship exists between *T*_½_ and Δ*G*_C_:

3 $ \Delta G_{C} = \Delta S(T_{\rm{½}} - T),  $

where all contributions to temperature-independent enthalpy to Δ*G*_C_ are absorbed in the term T_½_Δ*S*. Therefore, the opening probability of the thermo-gating channel can be restated as:

4 $ {P}_{\rm{o}\rm{p}\rm{e}\rm{n}}\left(T\right)=\left\{1 + \rm{e}\rm{x}\rm{p}\left[\frac{\left({T}_{\rm{½}}-T\right)\Delta S}{RT}\right]\right\}^{-1}. $

It is important to note that the temperature variable *T* appears both in the nominator and denominator of the exponential term. Thus, the opening probability as a function of *T* slightly differs from those depending on a temperature-independent variable (*e.g.,* the transmembrane voltage). However, the function still exhibits a common *S*-shaped curve around *T*_½_ (Fig. 2B). The independent parameters of the bistable thermo-gating model, *T*_½_ and Δ*S*, can be deduced by curve fitting, as long as the absolute probability curve can be measured experimentally. Let us assume that *P*_open_ can rise from approx. 0 to nearly 1 within a 10 °C range (Δ*T*) in the vicinity of *T*_½_ (~40 °C). Then, under conditions of |*T*_½_–*T*|$ \ll $*T* (*i.e.,* 10 K $ \ll $313 K), the following formula holds true:

5 $ \frac{{dP}_{\mathrm{o}\mathrm{p}\mathrm{e}\mathrm{n}}}{dT}{|}_{T={T}_{\rm{½}}}\Delta T\approx \frac{\Delta S}{4R}\frac{\Delta T}{T}\approx 1 , $

6 $ \Delta S\approx 4R\frac{T}{\Delta T}=125R , $

where the universal gas constant (*R*) is used as the unit of entropy. Furthermore, since Δ*S* and Δ*H* are proportional to each other for a given *T*_½_, Δ*H* can also be used to gauge the thermodynamic requirement for the thermo-gating, as attempted previously (Voets* et al.* 2004). In the above example, Δ*H* equals to 125*RT*_½_, which is roughly equivalent to the energy necessary to break a common covalent bond (~340 kJ/mol). In addition, as a point mutation is unlikely to disturb Δ*S* significantly, *T*_½_ of a thermo-sensor can be altered by inducing a change in Δ*H*. For instance, to design a mutational variant which is of an adjusted *T*_½_ from 40 to 30 °C, the Δ*H* of the mutant needs to be reduced by 4*RT* (*i.e.*, ΔΔ*H*$ \approx 125RT\left(\Delta T/T\right) $). In other words, removing only two hydrogen bonds from the resting state of the thermo-sensor is probably sufficient for inducing a 10 °C change in its middle-point temperature.

In case that *T*_½_ is higher than normal physiological temperature (*i.e.*, resting temperature), the channel opening process is called hyperthermic gating; otherwise, it is called hypothermic gating. When only considering channel types that are present in their close states at the resting temperature, TRPV1 serves as a representative of type-I hyperthermic gating channels; and TRPM8 belongs to the type-II hypothermic gating channel category. To ensure that a channel is able to work repeatedly, the conformational change associated with large |Δ*S*| must be reversible. Critically, the reversible entropy change associated with the thermo-gating channel does not violate the Second Law of thermodynamics, because the driving energy is derived from an external temperature reservoir. Nevertheless, such a conformational change may not be instantaneous. In fact, a slightly delayed denaturation (as well as refolding) process of the peptide chain can manifest itself as the capacity for short-lived memory. For instance, it was previously reported that when several repeated heat pulses are applied to cells expressing TRPV3, the apparent conductance of the channels gradually increases (Xu* et al.* 2002). It is plausible that after the previous heat pulse stopped, the once-opened channels may not immediately return to their close state due to incomplete refolding; therefore, the following pulse before the thermo-equilibrium is reached will result in more channels opening. In addition, *T*_½_ should remain well below the melting point temperature (*T*_m_) of the main part of the channel complex. Such thermostability requirements can be easily met in those channels that contain multi-domain subunits. The thermo-sensors that perform the gating function in thermo-TRP channels most likely are the soluble domains from the channel subunits and/or their oligomeric complex located in the cytosol.

In principle, the same temperature-control mechanism is prevalent in all types of bistable channels, as well as other functional membrane proteins. However, their *T*_½_ values do not necessarily appear in a range that is physiologically relevant. In these cases, the energy term −*T*Δ*S* is combined with other energy terms inside Δ*G*_C_. For example, −*T*Δ*S* is usually incorporated into the *V*_½_*Q* term of the voltage-gating channel without explicit discussion. In contrast, when thermo-gating mechanisms are considered for a given channel, *VQ* is commonly incorporated into the *T*_½_Δ*S* term, in order to highlight temperature variation as the dominant gating factor. In a more complex model, it is feasible to construct a joint opening probability equation for polymodal bistate channels that simultaneously implement thermo-, voltage-, ligand binding-, and membrane tension-gating mechanisms (Zhang 2021). Within a unified thermodynamic framework, the bistable-model hypothesis discussed above provides a satisfactory simplification for the thermo-gating mechanism, as long as the transition with the steepest temperature dependence is rate limiting. The existence of such a mechanism warrants further investigation, either to be verified or falsified experimentally. Such experiments may include thermo-denaturation and mutagenesis studies on channel properties, among others. Until then, we propose that our model serves as the best possible explanation for the temperature-sensing mechanism of thermo-gating channels.

## Conflict of interest

Xuejun C. Zhang and Zhuoya Yu declare that they have no conflict of interest.
